# Harnessing Multi-Omics and Predictive Modeling for Climate-Resilient Crop Breeding: From Genomes to Fields

**DOI:** 10.3390/genes16070809

**Published:** 2025-07-10

**Authors:** Adnan Amin, Wajid Zaman, SeonJoo Park

**Affiliations:** Department of Life Sciences, Yeungnam University, Gyeongsan 38541, Republic of Korea; adnan.amin@yu.ac.kr (A.A.); wajidzaman@yu.ac.kr (W.Z.)

**Keywords:** multi-omics integration, predictive modeling, climate-resilient crops, genotype to phenotype, digital phenotyping, genotype-by-environment interaction, machine learning, genomic selection

## Abstract

The escalating impacts of climate change pose significant threats to global agriculture, necessitating a rapid development of climate-resilient crop varieties. The integration of multi-omics technologies—such as genomics, transcriptomics, proteomics, metabolomics, and phenomics—has revolutionized our understanding of the intricate molecular networks that govern plant stress responses. Coupled with advanced predictive modeling approaches such as machine learning, deep learning, and multi-omics-assisted genomic selection, these integrated frameworks enable accurate genotype-to-phenotype predictions that accelerate breeding for augmented stress tolerance. This review comprehensively synthesizes the current strategies for multi-omics data integration, highlighting computational tools, conceptual frameworks, and challenges in harmonizing heterogeneous datasets. We examine the contribution of digital phenotyping platforms and environmental data in dissecting genotype-by-environment interactions critical for climate adaptation resilience. Further, we discuss technical, biological, and ethical challenges, encompassing computational bottlenecks, trait complexity, data standardization, and equitable data sharing. Finally, we outline future directions that prioritize scalable infrastructures, interpretability, and collaborative platforms to facilitate the deployment of multi-omics-guided breeding in diverse agroecological contexts. This integrative approach possesses transformative potential for the development of resilient crops, ensuring agricultural sustainability amidst increasing environmental volatility.

## 1. Introduction

Climate change has been increasingly threatening global food security through the exacerbation of abiotic stresses such as drought, heat, salinity, and flooding, which substantially reduce crop productivity and yield stability worldwide [1,2]. The frequency and severity of these environmental challenges are escalating, thereby compromising the resilience of many staple crops essential for human nutrition. Such stress conditions impose complex physiological and molecular constraints on plant growth and development, triggering dynamic alterations across multiple biological layers [3]. The pressing need to maintain agricultural productivity amid changing climate conditions drives the search for innovative strategies to enhance crop resilience beyond the capabilities of conventional breeding.

Conventional breeding techniques, while foundational to crop improvement over the past century, often struggle to keep pace with the rapid environmental changes and the complex genetic architecture governing stress tolerance traits [4]. These methods rely heavily on phenotypic selection and relatively simple marker-assisted approaches, which are constrained by limited throughput, the low resolution of polygenic trait architecture, and prolonged breeding cycles [5]. Moreover, environmental variability frequently masks genotype performance, hindering the identification and fixation of desirable alleles [6]. The polygenic and environmentally sensitive attributes of climate-adaptive traits necessitate a paradigm shift toward more precise, data-driven breeding frameworks capable of dissecting trait complexity at the molecular, biochemical, and physiological levels [7].

The integration of systems biology approaches, especially multi-omics methodologies, has transformed our understanding of plant responses to environmental stressors. Genomics, transcriptomics, proteomics, metabolomics, and phenomics collectively facilitate the comprehensive profiling of the molecular and phenotypic landscapes, elucidating the complex networks that regulate stress adaptation [8]. Advances in high-throughput sequencing, mass spectrometry, and imaging technologies generate vast, multidimensional datasets that reflect dynamic biological processes across scales [9]. Concurrent advancements in computational biology and machine learning offer powerful tools to integrate heterogeneous datasets, uncover hidden patterns, and build predictive models that link genotype to phenotype [10,11]. These integrative frameworks augment our ability to discern key regulators, biomarkers, and pathways that contribute to climate resilience, thereby accelerating the breeding pipeline.

Predictive modeling approaches, leveraging both classical machine learning algorithms and deep learning architectures, utilize multi-omics datasets to predict and model complex traits such as yield stability and stress tolerance under diverse environments [12,13]. The integration of digital phenotyping with environmental covariates refines the accuracy of such models, enabling the capture of genotype-by-environment interactions that majorly influence plant performance [14]. These data-driven strategies reduce the reliance on trial-and-error practices and enable breeders to prioritize candidate genotypes with enhanced accuracy. Moreover, multi-omics-informed genomic selection frameworks provide extensive insights by integrating genetic markers with transcriptomic and metabolic profiles, thereby increasing the prediction accuracy of breeding values [15].

This review synthesizes current advances in multi-omics technologies and predictive modeling as applied to climate-resilient crop breeding. It critically evaluates the attributes of individual omics platforms and their integration strategies, emphasizing computational tools and challenges associated with multi-layer data integration. The contributions of machine learning and deep learning in phenotype prediction and genomic selection are examined, alongside the incorporation of high-throughput digital phenotyping and environmental data to tackle genotype-by-environment interactions. Additionally, this review addresses the technical, biological, and ethical challenges facing multi-omics-guided breeding and proposes practical strategies for building robust, scalable breeding pipelines. This comprehensive synthesis provides a valuable resource for researchers and breeders aiming to harness multi-omics and predictive frameworks to sustainably improve crop resilience in the context of global climate change.

## 2. The Multi-Omics Toolkit in Plant Stress Biology

Research on plant stress responses and adaptation has been greatly enhanced by the use of multi-omics technologies, which collectively provide a systems-level perspective on how plants perceive, respond, and adapt to environmental stressors. Each omics layer, from genomics to phenomics, elucidates distinct facets of plant biology, offering complementary insights that collectively construct a comprehensive picture of stress adaptation mechanisms [8]. Genomics elucidates the genetic basis and structural variation responsible for stress resilience, whereas transcriptomics uncovers the dynamic regulation of gene expression and alternative splicing patterns in response to stress conditions [16]. Proteomics sheds light on functional proteins and post-translational modifications that mediate rapid cellular responses, while metabolomics profiles the biochemical environment that reflects stress-induced metabolic reprogramming [17]. Phenomics quantifies observable traits using advanced imaging and sensor technologies, facilitating the identification of direct associations between molecular changes and phenotypic outcomes [18]. The integration of these layers informs the development of predictive models and guides the breeding of crops with augmented stress resilience.

### 2.1. Genomics and Pan-Genomics in Crop Stress Adaptation

**Figure 1:**
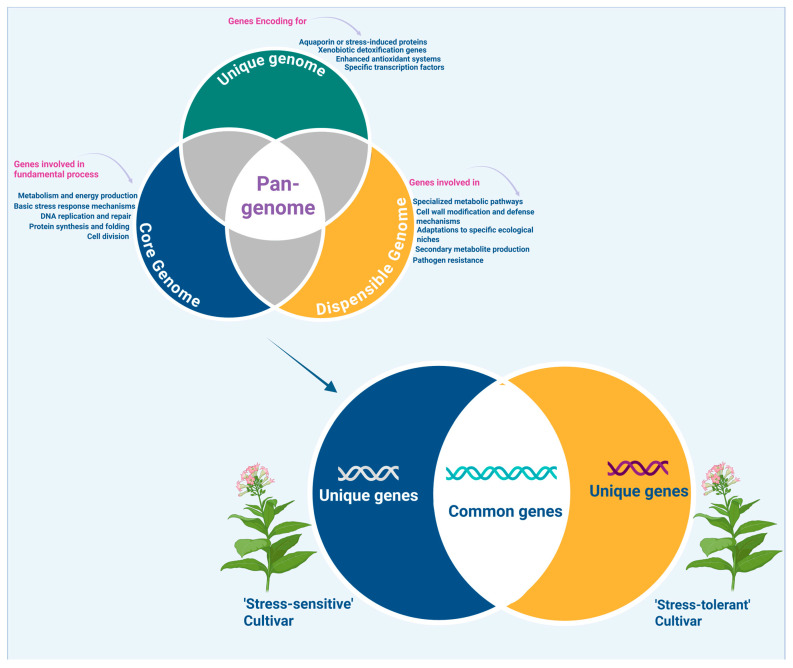
Schematic representation of pan-genome structure (core, dispensable, and unique genes) across ’stress-tolerant’ and ’stress-sensitive’ cultivars.

Genomics forms the foundation for understanding plant stress adaptation through the identification of DNA-level variations such as single-nucleotide polymorphisms (SNPs), copy number variations, and larger structural rearrangements [19]. Traditional single-reference genome assemblies capture only a fraction of the genetic diversity present within a species, thereby constraining the identification of critical (and in many occasions genotype- or environment-specific) adaptive loci [20]. To overcome these shortcomings, the pan-genome concept integrates multiple genomes from diverse accessions to categorize genes into core genes shared by all individuals, dispensable genes present in some but not all genotypes, and unique genes specific to particular genotypes [21]. This approach unveils hidden genetic variation, particularly in dispensable and unique regions that often harbor stress-responsive genes not present in reference genomes [22]. These variants play crucial roles in conferring tolerance to environmental stresses such as drought, salinity, and temperature extremes [23]. Genome-wide association studies (GWASs) and quantitative trait locus (QTL) mapping, utilizing dense SNP and structural variant data, enable the identification of genomic regions associated with stress tolerance traits, accelerating marker-assisted breeding [24]. This approach expands the genetic toolkit available for crop improvement beyond conventional limits (Figure 1).

### 2.2. Transcriptomics and Alternative Splicing Analyses Under Stress

Transcriptomic analyses reveal how plants orchestrate gene expression changes to adapt to stress conditions [25]. RNA sequencing (RNA-seq) provides a comprehensive analysis of genome-wide expression, identifying differentially expressed genes involved in stress signaling, defense, and repair [26]. Alternative splicing contributes to transcriptome plasticity by generating multiple transcript isoforms from individual genes, diversifying the proteome, and modulating stress responses [27]. Regulatory non-coding RNAs, such as microRNAs and long non-coding RNAs, mediate additional control by fine-tuning gene expression at the post-transcriptional or epigenetic level [28]. Cutting-edge single-cell RNA sequencing (scRNA-seq) technologies have begun to unravel cell-type-specific transcriptional responses, uncovering heterogeneity in stress adaptation at the cellular level [29]. Extensive transcriptomic datasets are now available for numerous plant species and stress conditions, providing a rich resource for comparative genomics and functional studies. Table 1 compiles key plant transcriptomic datasets, detailing the platforms and stress types, offering researchers a valuable guide for selecting suitable datasets for integrative analyses.

### 2.3. Proteomics and Post-Translational Modifications

**Figure 2:**
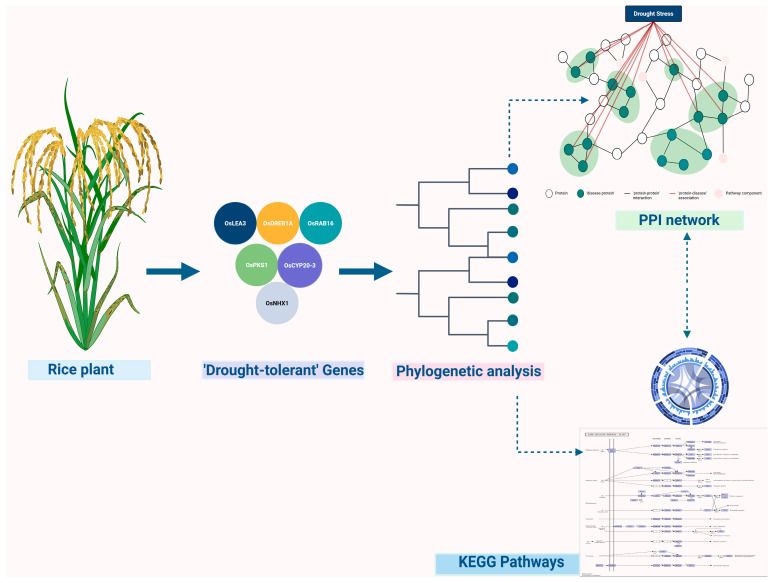
Protein–protein interaction (PPI) network and signaling pathway analysis under drought stress in rice.

Proteomics extends beyond transcriptional regulation to measure the abundance, modifications, and interactions of proteins carrying out cellular functions [39]. In response to stress, plants extensively remodel their proteomes, modifying enzyme activities, structural proteins, and regulatory components [40]. Post-translational modifications (PTMs) such as phosphorylation, ubiquitination, and acetylation act as molecular switches that rapidly adjust protein function, localization, and turnover, enabling plants to fine-tune their responses to environmental stressors [41,42]. Advances in mass spectrometry have enhanced the sensitivity and resolution of proteomic analyses, facilitating the comprehensive profiling of PTMs and protein networks. The ’protein–protein’ interaction (PPI) analyses reveal clusters of proteins that act cooperatively in signaling pathways and stress response complexes [43]. The PPI network shown in Figure 2, generated under drought stress conditions in rice, exemplifies how proteomics uncovers key regulatory hubs that could be targeted for enhancing stress resilience through molecular breeding.

### 2.4. Metabolomics and Stress-Induced Biochemical Pathways

Metabolomics complements genomics and proteomics by profiling small molecules that reflect the physiological and biochemical state of plants under stress [8]. Both primary metabolites, such as sugars, amino acids, and organic acids, and secondary metabolites, including phenolics, alkaloids, and terpenoids, play vital roles in osmoprotection, antioxidant defense, signaling, and stress tolerance [44]. Advanced analytical platforms such as liquid chromatography–mass spectrometry (LC-MS), gas chromatography–mass spectrometry (GC-MS), and nuclear magnetic resonance (NMR) facilitate metabolite detection and quantification with high sensitivity. By integrating metabolomic data with transcriptomic and proteomic profiles, metabolic pathways perturbed during stress can be reconstructed, enabling the identification of key biomarkers predictive of tolerance [8]. Table 2 summarizes representative metabolite classes, their corresponding analytical techniques, and their relevance to various stress responses, serving as a valuable reference point for experimental design and interpretation.

### 2.5. Phenomics for Stress Tolerance Trait Quantification

**Figure 3:**
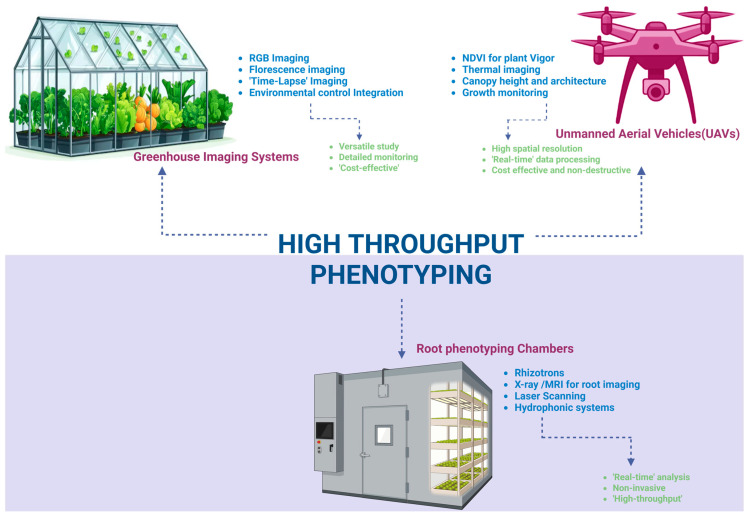
Schematic representation of a high-throughput phenotyping setup including UAVs, greenhouse imaging, and root phenotyping chambers with their key features and advantages.

Phenomics harnesses advanced imaging and sensor technologies to capture complex phenotypic traits associated with stress tolerance [53]. High-throughput phenotyping (HTP) platforms utilize instruments and equipment including unmanned aerial vehicles (UAVs), multispectral and thermal cameras, fluorescence imaging, and root phenotyping chambers to measure traits such as the canopy temperature, leaf water content, stomatal conductance, chlorophyll fluorescence, leaf area, and root system architecture [54]. These non-destructive, high-resolution measurements facilitate the temporal and spatial monitoring of stress responses across many genotypes in both controlled and field environments [55]. Integrating phenotyping information with molecular data enhances the understanding of genotype–phenotype relationships and improves the accuracy of predictive breeding models [53]. Figure 3 illustrates a typical HTP setup combining UAVs, greenhouse imaging, and root phenotyping, highlighting the multifaceted nature of phenomic data acquisition.

## 3. Strategies for Multi-Omics Data Integration

Multi-omics data integration is crucial to elucidating complex traits like plant stress responses. Combining genomics, transcriptomics, proteomics, metabolomics, and phenomics outputs provides a multidimensional view of biological systems but requires sophisticated computational and statistical methods to effectively unify heterogeneous datasets [56]. The complexity arises from both the extensive volume and diversity of data types and from the biological intricacies of signaling pathways, gene regulation, and environmental interactions that these data represent [57]. Successfully integrating these layers uncovers hidden relationships and causal links, enabling improved predictive models of phenotype from genotype [58]. For example, the integration of transcriptomic and metabolomic data in rice under drought stress has revealed the coordinated regulation of abscisic acid biosynthesis and osmolyte accumulation, linking gene expression changes to metabolic adaptation [59]. Such integrative approaches are crucial for identifying robust biomarkers and key regulators for climate-resilient breeding.

### 3.1. Conceptual Frameworks for Omics Integration

Strategies for integrating multi-omics data can be broadly categorized into horizontal, vertical, and diagonal approaches [60]. Horizontal integration merges datasets of the same omics data type collected under different conditions or from diverse populations, thereby improving reproducibility and robustness [61]. For example, integrating transcriptome datasets from multiple drought stress experiments in maize enhances the reliability of the identified stress-responsive genes by emphasizing consistently regulated transcripts [62]. Vertical integration involves linking multiple omics layers measured on the same samples, capturing biological cascades from DNA variation extending to RNA, a protein, and metabolite dynamics [63]. This approach can elucidate regulatory hierarchies; for instance, an integrative analysis in Arabidopsis linking SNPs, gene expression, protein abundance, and metabolic shifts under salt stress unraveled novel transcription factors governing tolerance [8,64]. Diagonal integration uses multi-omics data from distinct yet related samples connected by shared phenotypes or pathways, allowing inference when fully matched datasets are unavailable [65]. These conceptual models guide experimental design and computational workflows, with the choice depending on the study objectives and available data.

**Figure 4:**
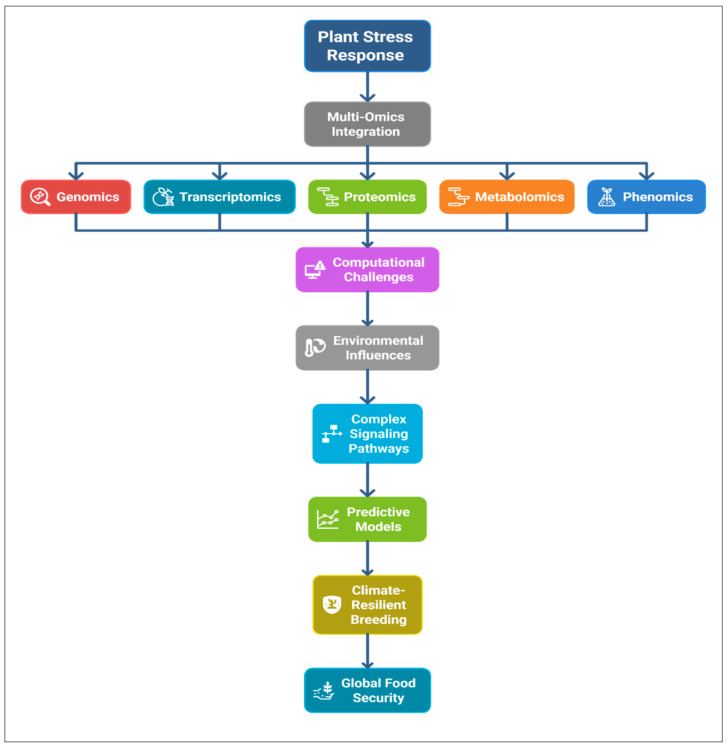
Layered integration framework illustrating genome–transcriptome–proteome–metabolome flow into phenotype prediction.

Beyond conceptual definitions, integration frameworks often employ network-based and multivariate statistical approaches. Horizontal and vertical models can be complemented by pathway and gene regulatory network reconstruction, facilitating biological interpretation [66]. A recent study integrated co-expression and protein interaction networks with metabolite correlation graphs to dissect drought tolerance in wheat, highlighting cross-omics modules predictive of yield stability [67]. The hierarchical structure of biological systems supports layered integration wherein outputs from one omics level inform analyses at another, thereby iteratively refining candidate genes and metabolite lists [68]. Visualizing these interactions through integrative frameworks, exemplified in Figure 4, elucidates the convergence of individual omics layers toward phenotype prediction.

### 3.2. Integration Tools and Data Fusion Platforms

A variety of computational tools have been developed to enable the robust integration of multi-omics datasets [69]. The mixOmics R package offers a comprehensive toolkit for multivariate analysis, encompassing canonical correlation analysis (CCA) and partial least squares (PLS), supporting the supervised and unsupervised integration of heterogeneous data [70]. For example, mixOmics facilitated the joint analysis of transcriptome and metabolome datasets in rice under cold stress, revealing molecular signatures associated with tolerance [71]. MOFA+ employs a Bayesian latent factor model that effectively manages missing data and detects both shared and dataset-specific variation across omics types [72]. It has been implemented in the integration of proteomics and metabolomics datasets in maize under nitrogen deficiency, revealing distinct, yet complementary, biological responses [73].

Network-based tools such as iOmicsPASS integrate multi-omics features with phenotype data to prioritize biomarkers and functional modules, useful in crop trait prediction [74]. Weighted Gene Co-expression Network Analysis (WGCNA) constructs modules based on correlated expression patterns and has been expanded to incorporate proteomics and metabolomics data, facilitating the identification of cross-omics trait-associated clusters [75]. DIABLO, part of the mixOmics suite, focuses on supervised integration for biomarker discovery and classification [76]. Table 3 delineates a comparison of these tools, highlighting their supported omics types, statistical methodologies, typical use cases, and limitations such as computational demand or sensitivity to data sparsity.

### 3.3. Technical Challenges in Omics Fusion

Notwithstanding progress, the integration of multi-omics data continues to be hindered by technical and biological challenges. The heterogeneity of data types including discrete SNP genotypes, continuous expression values, and semi-quantitative metabolite abundances complicates normalization and scaling [84]. For instance, transcriptomics datasets frequently contain zero-inflated count data requiring specialized models, whereas metabolomics data may have batch effects and varying detection limits [85]. Missing data is prevalent due to incomplete sample profiling or technical failures; imputation methods exist but may introduce biases. The disparity in data dimensionality, characterized by one omics layer significantly exceeding the number of features in others, can result in dominance during integrative analyses, thereby obscuring weaker yet critical signals [69].

Biological complexity further complicates interpretation due to nonlinear relationships, pleiotropy, and epistasis, which hinder model construction [86]. Environmental effects introduce noise, especially in field-collected phenomics data [87]. Computational demands for processing high-dimensional multi-omics datasets require high-performance infrastructure, limiting accessibility. Emerging strategies such as transfer learning and federated data analysis seek to address certain limitations. The validation of integrative models using independent datasets or experimental assays remains essential but is often overlooked [88]. Addressing these challenges is critical for the implementation of multi-omics data integration as a routine tool in breeding programs.

## 4. Predictive Modeling for Trait Selection

The increasing availability of high-dimensional multi-omics datasets has revolutionized plant breeding by enabling the development of sophisticated predictive models that link genotypes to complex phenotypes [89,90]. These models harness machine learning (ML) and deep learning (DL) algorithms to identify nonlinear interactions and hidden patterns across genomic, transcriptomic, proteomic, metabolomic, and phenotypic layers [91]. The objective is to predict traits such as yield, stress tolerance, and disease resistance with higher accuracy, reducing reliance on laborious field trials and accelerating breeding cycles [89]. Multi-omics integration enriches the input feature space by incorporating regulatory and metabolic information beyond genomic variants alone, thereby addressing the issue of missing heritability that constrains classical genomic selection [92]. In recent years, predictive modeling frameworks have evolved to incorporate environmental variables, longitudinal phenotyping data, and genotype-by-environment (G × E) interactions, enhancing the real-world applicability of predictions [93].

### 4.1. Machine Learning Algorithms for Trait Prediction

**Figure 5:**
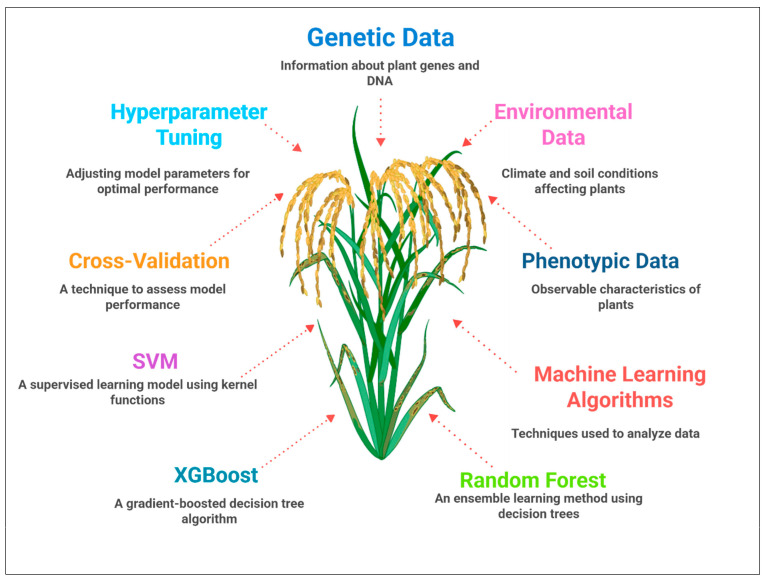
A detailed overview of different machine learning model training with multi-omics features for trait prediction.

Machine learning algorithms, such as Random Forest (RF), Extreme Gradient Boosting (XGBoost), and Support Vector Machines (SVMs), are extensively implemented in plant trait prediction due to their flexibility and ability to model complex nonlinear relationships [94]. RF constructs an ensemble of decision trees, each trained on bootstrapped samples, which collectively improve prediction robustness and handle high-dimensional multi-omics inputs with minimal parameter tuning [95]. To this end, Wu et al. (2024) applied RF models that integrated SNP genotypes and transcriptomic markers in maize subjected to drought conditions, achieving a predictive R^2^ of 0.72 for grain yield, substantially outperforming single-omics models [96]. XGBoost, which sequentially optimizes decision trees to minimize error, can efficiently handle heterogeneous datasets and missing values [97] and was implemented to integrate genomic, metabolomic, and environmental data in rice, improving yield prediction accuracy. SVMs, relying on kernel methods, classify complex trait outcomes by mapping data into high-dimensional spaces. Liu et al. (2024) employed SVMs with multi-omics data to predict wheat disease resistance with higher accuracy [98]. These models typically necessitate extensive cross-validation and hyperparameter tuning to prevent overfitting and maximize generalizability. Their interpretability can be enhanced through the utilization of feature importance metrics, aiding breeders in prioritizing candidate loci or biomarkers. Figure 5 depicts a typical ML pipeline integrating multi-omics features to train models predicting phenotypes such as yield under drought.

### 4.2. Deep Learning for Omics-Guided Genotype-to-Phenotype Modeling

Deep learning enhances predictive capabilities by autonomously acquiring hierarchical representations from raw multi-omics data, excelling in capturing spatial, temporal, and nonlinear dependencies. Convolutional neural networks (CNNs), originally developed for image recognition, have been repurposed for genomic sequence analysis and spatial phenotyping data [99]. Liang et al. [100] employed CNNs to analyze soybean genomic and UAV-derived image data, thereby increasing stress tolerance prediction accuracy by 15% compared to traditional ML. Recurrent neural networks (RNNs), especially Long Short-Term Memory (LSTM) models, are well-suited for handling sequential data such as time-series transcriptomics and phenomics, capturing dynamic stress response trajectories [101]. Autoencoders execute unsupervised dimensionality reduction and denoising of noisy, high-dimensional omics datasets, facilitating the extraction of key features that improve downstream phenotype prediction [102]. Recent advances integrate multi-modal deep learning architectures that jointly process heterogeneous omics and imaging data, exemplified by maize yield prediction models integrating genomics, metabolomics, and multispectral images [103]. Table 4 summarizes performance metrics including the R^2^, root mean square error (RMSE), and accuracy of various DL models applied to plant omics, highlighting their promise and current limitations related to model interpretability and data requirements.

### 4.3. Genomic Prediction Enhancement with Multi-Omics Layers

Genomic selection (GS) predicts breeding values using genome-wide marker data and has emerged as a standard approach for complex trait improvement [109]. Nevertheless, GS models traditionally rely on genomic data alone, potentially overlooking intermediate molecular phenotypes that mediate gene-to-trait associations [110]. The integration of transcriptomic and metabolomic data improves GS efficacy by providing functional context and capturing regulatory variation influencing traits. For example, RNA-seq expression profiles were incorporated into GBLUP models for maize drought tolerance, resulting in a reported increase of over 5% in prediction accuracy compared to the use of SNP markers alone [111]. Similarly, it was observed that incorporating metabolite profiles into genomic data improved wheat grain protein content predictions, aiding selection for nutritional quality [112]. These models utilize Bayesian approaches like BayesA or GBLUP and increasingly adopt kernel-based and machine learning extensions to accommodate multi-omics inputs. Challenges include the need for large, matched multi-omics datasets, computational costs, and the modeling of genotype-by-environment interactions [113]. Nonetheless, multi-omics-assisted GS represents a powerful strategy for enhancing predictive breeding accuracy and accelerating the selection of climate-resilient crop varieties.

## 5. Digital Phenotyping and Environmental Interfacing

Digital phenotyping is rapidly transforming plant breeding by providing precise, high-throughput, and non-destructive measurements of plant traits across multiple scales, from individual organs to entire crop fields [54,114]. These technologies facilitate the continuous monitoring of plants under realistic environmental conditions, capturing the temporal dynamics of stress responses [115]. As climate change increases environmental variability, integrating digital phenotyping data with omics and environmental sensing has become crucial for the dissection of genotype-by-environment (G × E) interactions and the development of more predictive and robust breeding models [116]. Digital phenotyping platforms encompass aerial drones, ground sensors, and root imaging systems that collectively enable the comprehensive evaluation of physiological and morphological traits associated with stress tolerance [117], directly supporting precision breeding efforts.

### 5.1. High-Throughput Phenotyping Platforms

High-throughput phenotyping (HTP) platforms have evolved to combine multiple sensing modalities, enabling detailed trait measurements at unprecedented speed and scale. UAVs equipped with multispectral, thermal infrared, and RGB cameras can rapidly assess canopy temperature, leaf area index, vegetation indices such as NDVI, and indicators of stress such as wilting or chlorosis symptoms across large field trials [118,119]. These aerial platforms can capture spatial heterogeneity and temporal changes in plant performance throughout the growing season. For example, thermal imaging has been successfully implemented to uncover heat- and drought-tolerant wheat genotypes by measuring canopy temperature depression, a proxy for transpiration efficiency [120].

In conjunction with UAVs, ground-based sensors such as chlorophyll fluorometers are employed to measure photosynthetic efficiency, providing insights into stress-induced damage at the leaf level [121]. Infrared gas analyzers can estimate stomatal conductance and transpiration rates, which are major indicators for extrapolating water use efficiency under drought [122]. Root phenotyping systems, including X-ray computed tomography and rhizotron imaging, can be implemented to accurately quantify belowground traits like root length, branching, and depth, which are critical for nutrient and water acquisition but traditionally difficult to measure [123]. These high-resolution datasets, collected non-invasively and repeatedly, enable dynamic phenotyping that captures the progression of stress responses, providing extensive temporal datasets essential for linking to underlying stress adaptation molecular mechanisms. Table 5 details key stress-related traits measurable by different HTP technologies and their corresponding sensing methods.

### 5.2. Modeling G × E Interactions in Predictive Breeding

Genotype-by-environment (G × E) interactions profoundly affect plant performance, especially under fluctuating climate conditions [133]. Traditional statistical models like Additive Main Effects and Multiplicative Interaction (AMMI) and genotype plus genotype-by-environment interaction (GGE) biplots have been fundamental in assessing G × E by partitioning variance components and visualizing genotype stability and adaptability across environments [134,135]. These approaches discern genotypes exhibiting broad adaptability or specific niche suitability. Recent advancements have integrated spatial statistical models and machine learning algorithms to better capture environmental gradients and local site effects, thereby improving prediction accuracy. For instance, Tesfaye et al. (2016) applied spatial mixed models incorporating soil moisture and temperature sensors, increasing the accuracy of drought tolerance predictions in maize breeding [136].

Biplots generated by GGE (genotype + genotype × environment interaction) analysis serve as a powerful and intuitive tool for visualizing genotype performance across diverse environments and traits, offering critical insights into genotype–environment interactions [137,138]. Such biplots plot genotypes (typically different breeding lines or varieties) against multiple traits (such as yield, disease resistance, drought tolerance, etc.) under diverse environmental conditions, allowing for the identification of patterns in genotype performance. By assessing the performance of various genotypes across diverse environments, breeders can identify those that demonstrate stability and superior traits under specific environmental stresses [139]. For instance, some genotypes may perform exceptionally well in drought-prone areas but show poor performance in regions with higher rainfall, while others may demonstrate resilience to both drought and heat stress [140]. This visual representation facilitates the selection of genotypes with superior traits, allowing breeders to prioritize those that are best suited for specific target environments. Furthermore, GGE biplots can contribute to the delineation of genotype × environment interactions by separating the effects of the genotype from those of the environment, providing a clearer picture of how genetic factors and environmental conditions jointly influence trait expression [141]. Through such analyses, breeders can make more informed decisions, targeting genotypes with the greatest yield stability potential, stress resistance, and adaptability [142], thereby optimizing breeding programs aimed at improving crop performance under fluctuating environmental conditions. The biplot’s intuitive design facilitates the communication of complex breeding data and results, thereby improving collaboration and decision-making in crop improvement strategies [143]. Moreover, integrating these models with omics-informed predictive frameworks establishes a holistic selection system that accounts for both genetic potential and environmental responsiveness.

### 5.3. Integrating Climate and Omics Data

The integration of high-resolution environmental data with omics and high-throughput phenotyping data represents a frontier in predictive breeding [116]. Satellite remote sensing provides spatially explicit data on climatic variables such as temperature, precipitation, soil moisture, and vegetation health at regional scales, whereas Internet of Things (IoT) sensors installed in experimental fields can record and transmit fine-scale microclimatic conditions including humidity, solar radiation, and wind speed [144,145]. Integrating these environmental data streams with genomic, transcriptomic, metabolomic, and phenomic profiles enables the formulation of environment-aware selection indices and predictive models that reflect real-world growing conditions.

To this end, Singh et al. [146] integrated hourly temperature and humidity data with multi-omics and phenomics datasets in wheat, improving the prediction of heat stress tolerance and facilitating the selection of genotypes tailored for specific climatic niches. This approach supports dynamic breeding pipelines that adapt to spatial and temporal environmental variability, crucial for future-proofing crops against climate change. Challenges remain in the standardization of data formats, the management of extensive and heterogeneous datasets, and the development of computational frameworks that can concurrently model genotype, phenotype, and environmental features [147]. Nevertheless, this integrative approach paves the way for precision breeding strategies that maximize genetic gains while ensuring stability under variable climates.

## 6. Challenges and Opportunities

While multi-omics integration and predictive modeling have immense potential to accelerate climate-resilient crop breeding, several significant challenges limit their full realization and implementation. Such challenges encompass computational bottlenecks, biological complexities, data governance, and infrastructure gaps [148]. Addressing these obstacles requires interdisciplinary efforts involving computational scientists, plant biologists, breeders, and policymakers. Despite these challenges, innovative solutions and collaborative frameworks offer promising pathways to harness multi-omics for practical, scalable, and equitable crop improvement.

### 6.1. Computational and Data Integration Bottlenecks

One of the foremost challenges in multi-omics research lies in the computational infrastructure and methods required to process, store, and analyze vast heterogeneous datasets [149]. Scalability issues arise as omics datasets expand in both volume and complexity, demanding high-performance computing resources often inaccessible to many research groups. The heterogeneous nature of omics data ranging from discrete genomic variants to continuous transcript and metabolite abundance complicates normalization and harmonization [69]. Integrating RNA-seq counts, proteomic intensities, and metabolite concentrations requires careful statistical adjustments to ensure comparability and prevent biases in downstream analyses [63]. Moreover, batch effects, missing values, and data sparsity present substantial obstacles that can distort integrative models. Cloud computing platforms such as Terra, DNAnexus, and CyVerse have started to address these challenges by providing scalable environments, standardized workflows, and collaborative spaces [150]; however, adoption in plant sciences is sporadic.

Advanced algorithms and software tools tailored for multi-omics data integration also demonstrate limitations in handling large-scale datasets with missing or noisy data. The lack of standardized data formats and metadata annotation exacerbates interoperability issues, hindering data reuse and cross-study comparisons [151]. Table 6 outlines common computational bottlenecks associated with distinct omics layers—such as peak calling in metabolomics or variant calling in genomics—and highlights mitigation strategies including normalization methods, imputation techniques, and algorithmic advances. Addressing and resolving these computational challenges is critical to enabling reliable multi-omics integration and subsequent application in breeding programs.

### 6.2. Biological Interpretation and Trait Complexity

Multi-omics analyses are further complicated by the biological complexities inherent in plant stress responses. Numerous agronomically important traits exhibit pleiotropy, where a single gene affects multiple phenotypes, as well as epistasis, which entails interactions between genes that collectively influence traits [162]. Such nonlinear genetic architectures can obscure causal relationships when analyzed through traditional linear models. Moreover, a large portion of heritable variation, referred to as “missing heritability”, remains unexplained even after incorporating genomic data, suggesting that regulatory mechanisms, epigenetics, and gene–environment interactions are likely major influential factors [163]. Environmental variability, including microclimatic differences and soil heterogeneity, adds noise and complexity, challenging the interpretation of omics–phenotype associations.

The complexity of integrating multiple molecular layers increases the difficulty of biological interpretation; for instance, changes in transcript levels do not always translate directly into changes in protein or metabolite levels due to post-transcriptional and post-translational regulation [164]. Therefore, careful experimental design, rigorous statistical modeling, and functional validation are essential for the precise interpretation of multi-omics results. Systems biology approaches, including network reconstruction and causal inference, can help disentangle complex interactions but remain computationally intensive and dependent on high-quality data [165]. Continued efforts to improve the biological interpretability of integrated models are vital to translating omics insights into actionable breeding strategies.

### 6.3. FAIR Data Principles and Ethical Considerations

**Figure 6:**
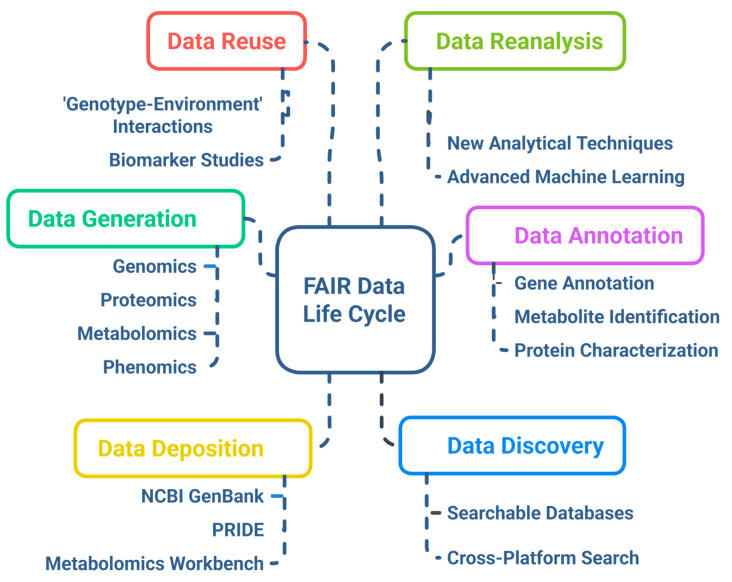
FAIR data life cycle implemented in multi-omics projects.

Ensuring that multi-omics data are Findable, Accessible, Interoperable, and Reusable (FAIR) is crucial for enhancing research impact and fostering reproducibility. Open data repositories such as NCBI GEO, EMBL-EBI, and MetaboLights facilitate broad access, but many datasets lack standardized metadata or consistent formatting, limiting integration [166]. The adoption of FAIR principles facilitates improved data sharing among institutions and bolsters global collaborative breeding initiatives [167]. However, challenges related to intellectual property rights (IPRs), especially in publicly funded research and private breeding programs, create tensions between open science and proprietary interests. Equitable benefit sharing with indigenous communities and countries of origin for genetic resources is also a critical ethical consideration, as mandated by international agreements like the Nagoya Protocol [168]. Figure 6 depicts the FAIR data life cycle in multi-omics projects, highlighting stages from data generation and annotation through deposition, discovery, reuse, and reanalysis. The integration of ethical frameworks and data governance policies with FAIR practices will be key to fostering trust and ensuring that multi-omics advances inclusively benefit global agriculture. Efforts to establish clear guidelines, data stewardship roles, and transparent licensing models are underway but require wider adoption and harmonization.

### 6.4. Roadmap for Future Integration

The future of multi-omics-guided crop breeding depends on the establishment of global platforms, consortia, and open-source tools that democratize access and foster collaboration. International initiatives such as the Crop Ontology Consortium and the Wheat Initiative exemplify efforts to standardize trait definitions and data-sharing protocols, enhancing interoperability [169]. Cloud-based infrastructures that accommodate multi-omics datasets alongside scalable analysis pipelines reduce barriers for researchers and breeders, enabling real-time data integration and decision-making [170]. Additionally, open-source software frameworks promote transparency, community contributions, and reproducibility, thereby enabling continuous improvement and adaptation to emerging technologies [171].

Training the next generation of interdisciplinary scientists skilled in both computational and plant biology domains is crucial to sustain progress [172]. Integrative research models that connect molecular biologists, data scientists, breeders, and policy experts will accelerate translation from data to deployed improved varieties [12]. Establishing and implementing common standards for data formats, metadata annotation, and model reporting will further harmonize the field. Through coordinated efforts, multi-omics integration will evolve from an experimental approach to a routine, scalable component of climate-resilient breeding pipelines.

## 7. Conclusions

The integration of multi-omics technologies with advanced predictive modeling has revolutionized crop breeding, especially in the context of developing climate-resilient varieties. Through the integration of genomic, transcriptomic, proteomic, metabolomic, and phenomic data, unprecedented insights can be obtained regarding the molecular mechanisms and complex trait architectures underlying stress adaptation. Predictive models, including machine learning, deep learning, and multi-omics-informed genomic selection, leverage these integrated datasets to accurately predict phenotypic performance under variable environmental conditions. Such integrated approaches surpass traditional breeding methods by enabling the identification of robust biomarkers, the dissection of genotype-by-environment interactions, and the acceleration of selection cycles. Multi-omics and predictive analytics are collectively facilitating a paradigm shift from phenotype-based selection toward data-driven precision breeding, providing optimism for sustaining agricultural productivity amid increasing climate challenges.

Despite these advances, realizing the full potential of multi-omics-guided breeding requires the resolution of several key issues and obstacles. The standardization of data generation, processing, and integration protocols is essential to ensure reproducibility, comparability, and interoperability across studies and institutions. Establishing unified ontologies, metadata standards, and data-sharing frameworks will enable seamless aggregation and meta-analyses of heterogeneous datasets. The interpretability of predictive models is equally critical: breeders and biologists must be able to understand and trust the biological relevance of model predictions to make informed decisions. Advances in explainable AI and integrative network analyses are paving the way for more transparent models that link predictive features to functional biology. Furthermore, it is essential to prioritize scalability and practical applicability by developing computational tools and phenotyping platforms that are accessible, cost-effective, and deployable across various breeding programs worldwide, including in resource-limited settings.

Looking forward, the continued convergence of multi-omics technologies, environmental data integration, and predictive modeling will drive the next generation of climate-smart crop breeding. Collaborative international efforts to build open-source platforms, cloud-based infrastructures, and global consortia will democratize access to cutting-edge tools and data. Training interdisciplinary scientists capable of bridging computational biology, plant science, and breeding will be vital to sustain innovation. As these approaches mature, they are expected to deliver resilient crops tailored to specific agroecological niches, thereby enhancing food security and agricultural sustainability. Ultimately, the integration of comprehensive biological data with robust predictive models marks a new era of precision agriculture that meets the challenges that arise from a changing climate.

## Figures and Tables

**Table 1:** Key plant transcriptomic datasets under abiotic/biotic stress with platforms (RNA-seq, scRNA-seq) and stress types.

Plant Species	Platform	Stress Type(s)	Key Findings	References
*Arabidopsis thaliana*	RNA-seq	Abiotic (heat and dehydration)	Stress increased full-length transcript variants via exon skipping in the *SR45a* gene	[29]
*Arabidopsis thaliana*	RNA-seq	Abiotic	42% of intron-containing genes were alternatively spliced; stress shifted isoform ratios	[30]
*Populus trichocarpa*	RNA-seq + Iso-Seq	Drought, salt, and temperature	Differential intron retention and isoform ratio switching across tissues	[31]
*Cassava (Manihot esculenta)*	Isoform-Seq, ssRNA-seq, Degradome-seq	Cold and drought	Intron retention dominant; cold stress altered splicing regulators and triggered transcript decay	[32]
*Brassica napus*	RNA-seq	Cold, salt, dehydration, and ABA	357 genes showed alternative splicing; hub genes linked to stress tolerance pathways	[33]
*Capsicum annuum* (Pepper)	RNA-seq	Biotic (bacteria, virus, and oomycete)	4354 genes with stress-induced AS; 841.49 Gb data compiled from 425 samples	[34]
*Zoysia japonica*	RNA-seq	Cold	Dataset ZjRTD1.0 enables precise analysis of cold-induced splicing and co-regulation of isoforms	[35]
*Schrenkiella parvula*	Iso-Seq + RNA-seq	Salinity	Isoform diversity linked to salt tolerance; distinct isoform usage vs. *A. thaliana*	[36]
*Glycine max* (Soybean)	RNA-seq	Drought	Over 2000 genes alternatively spliced; splicing factors enriched under drought conditions	[37]
*Camellia sinensis* (Tea)	RNA-seq	Biotic (gray blight)	Splicing changes correlated with catechin biosynthesis; DASGs associated with disease defense	[38]

**Table 2:** Representative metabolite classes, detection techniques, and their involvement in stress adaptation and tolerance.

Metabolite Class	Key Examples	Detection Techniques	Stress Type(s)	Physiological Functions	References
Sugars	Glucose, sucrose, trehalose, and raffinose	GC-MS, LC-MS, and NMR	Drought, salt, and cold	Osmoprotection, energy source, and stress signaling	[45]
Amino Acids	Proline, glutamate, GABA, and tryptophan	GC-MS, LC-MS, and NMR	Osmotic, oxidative, and heat	Osmotic balance, ROS scavenging, and precursors to secondary metabolites	[46,47]
Organic Acids	Malate, citrate, and fumarate	GC-MS and NMR	Drought, cold, and metal	Central in TCA cycle, energy production, and pH regulation	[48]
Phenolics	Flavonoids, phenolic acids, and lignans	LC-MS/MS and NMR	Oxidative, UV, and drought	Antioxidant activity, UV protection, and defense	[46,47]
Alkaloids	Serpentine, tabersonine, and vinblastine	Targeted LC-MS	Biotic (pathogen and herbivory)	Defense compounds and anti-insect and anti-fungal agents	[45]
Terpenoids	Tanshinones and monoterpenes	LC-DAD-MS and GC-MS	Pathogen and drought	Antimicrobial properties, ROS modulation, and signaling	[46]
Fatty Acids	Linoleic acid and oleic acid	GC-MS and UPLC-QTRAP-MS	Heavy metal and cold	Membrane fluidity and precursors to signaling-related lipids	[49]
Sugar Alcohols	Mannitol and sorbitol	GC-MS and LC-MS	Salinity and drought	Osmoprotectants and ROS scavengers	[50]
Hormonal Intermediates	ABA precursors and auxin conjugates	LC-MS/MS	Drought, salinity, and temperature	Regulators of gene expression and stress adaptation	[51]
Shikimate Pathway Intermediates	Chorismate and phenylalanine	LC-MS and NMR	Drought and biotic stress	Links between primary and secondary metabolism; stress adaptation compound synthesis	[51]
Volatile Organic Compounds (VOCs)	Isoprene and linalool	GC-MS	Heat and biotic stress	Defense, communication, and thermotolerance	[52]

**Table 3:** Comparison of multi-omics integration tools: omics types, methods, and limitations.

Tool	Supported Omics Types	Statistical Methodology	Integration Approach	Data Handling	Visualization	Typical Use Case	Limitations	References
mixOmics	Transcriptomics, proteomics, metabolomics, microbiome, and epigenomics	PLS, CCA, sPLS, and DIABLO	Both (via different functions)	Handles missing data moderately well; requires scaling	Yes—heatmaps, networks, and correlation circles	Multi-omics classification, biomarker selection, and exploratory analysis	Sensitive to missing data; requires tuning; overfitting risk in small datasets	[70,77]
MOFA+	Any continuous omics: transcriptomics, epigenomics, proteomics, and metabolomics	Bayesian latent factor model	Unsupervised	Robust to missing values; normalizes data internally	Yes—factor plots and feature weights	Identification of shared and specific signals across -omics; sample stratification	Complex to interpret latent factors; high computational load for large datasets	[78]
iOmicsPASS	Transcriptomics, proteomics, metabolomics, and phenotypic data	Partial correlation networks + phenotype weighting	Supervised	Requires preprocessed complete matrices	Yes—modular network visualization	Disease/trait prediction; pathway–phenotype linkage	Phenotypic data needed; may struggle with very sparse networks	[79]
WGCNA	Primarily transcriptomics, extendable to proteomics/metabolomics	Weighted correlation-based network analysis	Unsupervised	Requires complete data; sensitive to outliers	Yes—dendrograms and module–trait heatmaps	Identification of co-expressed modules, hub genes, and module–trait associations	Not natively multi-omics; manual integration required	[80]
DIABLO (in mixOmics)	Multi-block omics: transcriptomics, metabolomics, and proteomics	Supervised sparse PLS	Supervised	Performs variable selection; handles moderate sparsity	Yes—sample plots and relevance networks	Supervised feature extraction; class-based biomarker identification	Requires high-quality labels; less effective in unsupervised scenarios	[70]
Multi-Omics Factor Analysis (MOFA)	Transcriptomics, metabolomics, and epigenomics	Matrix factorization via variational inference	Unsupervised	Missing data allowed; scalable	Yes—dimensional reduction plots	Discovery of hidden factors driving variation across omics	Needs large sample sizes for meaningful factors; interpretability issues	[81]
IntLIM	Transcriptomics + metabolomics	Linear modeling with interaction terms	Supervised	Focused on two-omics comparisons	No (basic plots only)	Tests for phenotype-dependent omics interactions	Limited to two omics types; limited data scaling options	[82]
JIVE	Any omics (continuous)	Joint and individual variation explained	Unsupervised	Missing data imputation not supported	Limited (basic singular-value plots)	Decomposition of joint vs. specific signals across datasets	Requires manual feature interpretation; basic statistical output	[83]

**Table 4:** Performance metrics of DL models applied in plant omics prediction tasks (R^2^, RMSE, and accuracy).

Model Type	Input Modalities	Task/Application	Performance Metrics	Key Strengths	Limitations	References
CNN (Convolutional Neural Network)	Genomic sequences + UAV-derived images	Soybean stress phenotype prediction	Accuracy increased 15% over baseline; R^2^ ~0.71	Captures spatial dependencies; combines omics and imagery	Requires large, labeled image datasets	[104]
RNN/LSTM	Time-series transcriptome + stress phenotype data	Temporal modeling of stress responses	RMSE: 0.15–0.20; accuracy up to 89%	Effective for dynamic, sequential data	Sensitive to time-gap variation; needs careful tuning	[105]
Autoencoder	High-dimensional omics (e.g., transcriptome and metabolome)	Dimensionality reduction + phenotype prediction	RMSE: 0.12–0.18; comparable to LSTM; R^2^ ~0.68	Denoises data; unsupervised feature extraction	Black-box interpretability; sensitive to latent dimension choice	[104]
Variational Autoencoder (VAE)	Genomics + imaging + environmental metadata	Multi-modal maize yield prediction	Accuracy: 85–90%; lower RMSE vs. linear models	Captures nonlinear joint distributions	Computational cost; sampling noise	[106]
Multi-modal DL Model	Genomics, metabolomics, and spectral imagery	Yield prediction under abiotic stress	R^2^: 0.78; RMSE: 0.11; accuracy: ~88%	Integrates diverse-omics and environmental data	Requires harmonized, co-measured datasets	[107]
Hybrid CNN+LSTM	Genomic images + temporal gene expression	Combined spatial-temporal modeling	Accuracy: 90.2%; R^2^ ~0.76; reduced overfitting compared to standalone models	Leverages strengths of both CNNs and RNNs	Higher model complexity	[108]

**Table 5:** Traits measurable by high-throughput phenotyping (HTP) platforms and associated technologies (e.g., canopy temperature via infrared sensor).

Trait Measured	HTP Platform/Modality	Sensor Type/Technique	Biological Relevance	References
Canopy temperature	UAV-based aerial thermal imaging	Thermal infrared cameras	Proxy for stomatal conductance and transpiration under heat/drought stress	[124]
NDVI (Normalized Difference Vegetation Index)	UAV multispectral imaging	Multispectral cameras (Red/NIR)	Indicator of photosynthetic activity and biomass	[125]
Chlorosis/leaf senescence	RGB + multispectral UAV	RGB and spectral indices (e.g., GNDVI and SAVI)	Visual cues of nutrient stress, senescence, and disease	[126]
Photosynthetic efficiency (ΦPSII)	Ground-based sensor	Pulse-amplitude-modulated (PAM) chlorophyll fluorometry	Captures efficiency of light reactions in photosynthesis under stress	[127]
Stomatal conductance (gs)	Portable leaf gas analyzers	Infrared gas analyzer (IRGA)	Measures gas exchange related to water loss and carbon uptake	[128]
Root system architecture	X-ray CT, rhizotrons, and Shovelomics	High-resolution 3D imaging or transparent interface	Essential for belowground trait monitoring under drought or nutrient stress	[129]
Canopy structure/LAI	UAV LiDAR + multispectral fusion	Light Detection and Ranging (LiDAR) + NDVI	Reflects total photosynthetic surface and canopy penetration	[130]
Water use efficiency (WUE)	UAV-based multispectral + ET modeling	NDVI-derived biomass + evapotranspiration estimates	Assesses yield relative to water use; key trait under water-limited conditions	[131]
Transpiration rate	Proximal thermal imaging in automated systems	Leaf surface temperature profiles over time	Indicates water loss dynamics and drought response	[132]
Plant height/growth rate	Time-lapse 3D LiDAR or stereo vision	Structure-from-motion (SfM) and laser range scanning	Non-invasive quantification of growth dynamics	[129]

**Table 6:** Omics-specific computational bottlenecks and mitigation strategies.

Omics Layer	Computational Bottleneck	Mitigation Strategy	Explanation	References
Genomics	Variant calling variability; batch-specific sequencing biases	Standardized pipelines (e.g., GATK); batch correction tools like reComBat	Ensures consistent variant detection across batches; tools like reComBat mitigate batch effects in heterogeneous datasets	[152]
Transcriptomics	Integration of RNA-seq datasets with missing samples and inconsistent coverage	Bayesian models (e.g., TiMEG); advanced imputation and joint modeling	Models like TiMEG handle partially missing transcript data without requiring full imputation, improving integration reliability	[153]
Proteomics	High frequency of missing values due to detection limits and instrumentation	Missing-value-tolerant frameworks (e.g., matrix dissection and no imputation)	Mitigates imputation biases by correcting only where data are present, maintaining statistical integrity	[154]
Metabolomics	Peak alignment errors; inconsistent quantification across batches	Transformation and normalization pipelines; batch-aware preprocessing	Careful preprocessing and normalization mitigate artifacts from instrumentation and sample prep variability	[155]
Epigenomics	Data sparsity; modality-specific biases (e.g., methylation depth vs. accessibility)	Cross-modal imputation; graph neural networks; contrastive learning	Emerging AI methods (e.g., SpaMosaic) reconstruct missing modalities and enable high-fidelity integration	[156]
Multi-omics (general)	Heterogeneous data types and missing modalities	Deep generative models (e.g., VAEs); multi-modal latent space integration	Variational autoencoders and other AI tools embed omics data into shared spaces, enabling joint analysis and imputation	[157,158]
All layers (meta-level)	Batch effects coupled with missing data (BEAMs: Batch-Effect-Associated Missing Values)	Hybrid methods accounting for batch and missingness (e.g., MultiBaC, BEAM-aware imputation)	MultiBaC exploits shared modalities across datasets to correct for lab-specific artifacts; BEAM-aware workflows prevent imputation bias	[159,160]
Single-cell omics	High sparsity and noise in individual cell profiles; multi-modal alignment	Product-of-Experts VAEs; latent space alignment and batch-aware learning	Enables robust integration across scRNA, ATAC, and protein modalities in sparse, high-dimensional data	[161]
